# Exploring New Frontiers: Innovations and Therapeutic Targets in Dermatology

**DOI:** 10.3390/ijms25158102

**Published:** 2024-07-25

**Authors:** Montserrat Fernández-Guarino, Andrés González-García, Asunción Ballester Martínez

**Affiliations:** 1 Dermatology Service, Hospital Universitario Ramón y Cajal, Irycis, 28034 Madrid, Spain; 2 Internal Service, Hospital Universitario Ramón y Cajal, Irycis, 28034 Madrid, Spain

The focus of dermatology has increasingly shifted towards exploring new and innovative approaches. Treatments have progressively targeted molecular pathways, moving away from the non-specific methods of the past decade, such as general immunosuppressants, keratinization regulators, or drugs that act more generally on various points. This significant advancement has been made possible through synergies between basic research and clinical dermatology, along with the development of studies that allow for the discovery of different pathways at the laboratory level. These discoveries serve not only for the development of new therapies [1] but also for a better understanding of dermatological diseases [2].

In this context, we have gathered authors who excel in this field to bring together these innovations in dermatological skin diseases and focused treatments. A total of 11 manuscripts are compiled in this Special Issue with the aim of presenting the situation of novel dermatology. 

Dermatology has witnessed remarkable advancements over the last decades, both in our understanding of the pathophysiology of skin diseases and in the development of innovative therapies. This editorial highlights recent progress in these areas, emphasizing the significance of ongoing research and innovation in the treatment of inflammatory dermatological conditions. As shown in Table 1, different areas of inflammatory skin diseases are covered. 

The field of dermatology has significantly benefited from breakthroughs in genetics and molecular biology, transforming our understanding of numerous skin diseases (Contributions 2 and 9). For instance, the application of knowledge concerning specific genetic aspects may facilitate the implementation of a personalized approach to the treatment of patients with severe psoriasis (9). Berna-Rico et al. found an influence of some polymorphisms in the response to anti-TNF-α therapy. However, pharmacogenetic and pharmacoeconomic studies, which aim to predict both effectiveness and toxicity, are scarce in the literature. Furthermore, advances in next-generation sequencing technologies have enabled the identification of genetic mutations responsible for susceptibility to bullous pemphigoid in patients receiving DPP4 inhibitors (2). These genetic insights allow for the development of personalized medicine, tailoring treatments to individual genetic profiles and improving patient outcomes. 

In addition to genetic research, the study of immune mechanisms in skin diseases has yielded significant findings. Conditions such as psoriasis and vitiligo have been shown to involve complex interactions between immune cells and skin cells. Understanding these interactions has led to the identification of key cytokines and signaling pathways, offering new avenues for targeted therapies. In the case of vitiligo, further targeted therapies are still necessary, but an understanding of immune pathogenesis in the skin and in the systemic immune system allows for future directions (4).

One of the most promising areas of advancement in dermatology is the development of biological treatments. Biologic drugs, such as interleukin inhibitors, have transformed the management of chronic skin conditions, including psoriasis and atopic dermatitis. These drugs specifically target molecules involved in the inflammatory process, thereby providing more effective and sustained relief compared to traditional therapies. The advent of Janus kinase (JAK) inhibitors represents a pivotal advance in dermatological therapeutics. JAK inhibitors block specific pathways involved in immune responses, offering a novel approach to the treatment of conditions such as alopecia areata and vitiligo. Clinical trials have demonstrated the efficacy of these drugs in reducing disease severity and improving patients’ quality of life. In other complex diseases, such as neutrophilic dermatoses (7), these therapies have achieved a reduction in clinical symptoms and a decrease in the frequency of flares, although some of them are used off-label.

Treatments targeting interferon I and, in particular, type I interferon receptors have been developed for the treatment of systemic lupus erythematosus (SLE). Anifrolumab, a fully human, IgG1κ monoclonal antibody to subunit 1 of type I interferon receptor, was licensed for the treatment of SLE in 2021. In a post hoc analysis of the phase III TULIP 1 and TULIP 2 randomized controlled trials, focusing on skin involvement in patients with SLE, anifrolumab produced earlier and greater control of mucocutaneous lesions than did the placebo, using CLASI-A, BILAG-2004, and SLEDAI-2K as activity indexes [3]. These promising results from the use of anifrolumab for skin involvement in SLE have yet to be confirmed in patients with cutaneous lupus erythematosus (CLE). Several case series of patients with CLE treated with anifrolumab with impressive results have also been recently published [4,5]. Nevertheless, the results of a phase III trial of anifrolumab in adults with chronic or subacute cutaneous lupus erythematosus that is currently about to start recruitment will shed light on the potential effect of anifrolumab for patients with CLE [6]. 

Besides anifrolumab, other new treatments are currently under development for CLE, such as litifilimab, a monoclonal antibody directed against blood dendritic cell antigen 2 (BDCA2), a surface cell receptor expressed exclusively on plasmacytoid dendritic cells. The binding of litifilimab to BDCA2 receptor inhibits the release of type I interferon and other inflammatory cytokines. The results from a phase II trial comparing litifilimab against a placebo in patients with CLE favored the effect of litifilimab, as indicated by the change in the CLASI-A score at 16 weeks [7], with acceptable tolerance.

Type I interferon elicits its proinflammatory effect in lupus erythematosus via the activation of molecular signals, including Tyrosine Kinase 2 (TYK2). An oral allosteric inhibitor of TYK2 already approved for the treatment of psoriasis, deucravacitinib, has also been studied for SLE. In a phase II trial including adult patients with active SLE, those who received deucravacitinib experienced greater CLASI 50 responses at 32 and 48 weeks than did those treated with placebo [8]. These promising results need to be confirmed in specific trials for CLE. With all these promising drugs on the near horizon, we might be witnessing an imminent paradigm shift for the management of CLE and SLE.

Non-melanoma skin cancer is the most frequent cancer in the general population; the treatment and prevention of precancerous lesions such as Actinic Keratosis are under continuous investigation, and new target therapies have been commercialized due to this continuous research [9]. Non-melanoma skin cancer target therapies are based on in vitro cell keratocyte cultures and models of the skin, as in Contributions 7 and 11. The focus going forward is on prevention strategies and protocols working with the immune system, like those for melanoma skin cancer (9).

Laser technologies have also seen significant improvements. Fractional laser treatments, which create micro-injuries in the skin to stimulate natural healing processes, have proven effective in treating scars, wrinkles, and pigmentation disorders. These minimally invasive procedures offer patients improved cosmetic outcomes with reduced downtime [10]. Other new techniques, such as capacitive resistive electrical transfer therapy, or CRET, based on radiofrequency, are also being studied to prove their therapeutic role in the treatment of skin fibrosis and scars (3). Other physical and light therapies are developing and increasingly applied in dermatology, such as the use of LEDs, photobiomodulation, ultrasound, electrostimulation, and magnetic fields (1). Newer trans-epidermal drug delivery systems have been developed to treat several skin diseases, both inflammatory and malignant conditions, to avoid exposure to systemic drugs and minimize adverse events [11].

Despite these advancements, several challenges remain in the field of dermatology. There is a growing need for multidisciplinary assessments of patients. With new knowledge, addressing the increasing treatment problems and associated infectious complications in these patients is becoming more and more complex. The future of dermatology lies in greater interdisciplinary collaboration. Integrating knowledge from immunology, microbiology, and bioengineering will drive the development of innovative and effective solutions, allowing the development of personalized therapies that will optimize clinical results while minimizing risks. For example, combining immunotherapy with regenerative medicine approaches could revolutionize the treatment of chronic wounds and scars. Additionally, artificial intelligence and machine learning can be harnessed to enhance diagnostic accuracy and personalize treatment plans based on vast datasets.

In summary, advances in our knowledge of the pathophysiology of and therapies for dermatological conditions have significantly transformed patient care, improving outcomes and quality of life in several scenarios. The dermatological community must continue to explore new frontiers and work closely together to address ongoing challenges and uncover new therapeutic opportunities.

## Figures and Tables

**Table 1:** Analysis of the published contributions in this Special Issue.

N#	Research Area	Focus	Type of Research
1	Wound healing	Physical therapies in chronic wounds and assisted scarring	Review
2	Blistering diseases Bullous pemphigoid	Molecular mechanism of drug-induced pemphigoid	Review
3	Keloids and hypertrophic scars	Basic research on the potential mechanism of RF anti-fibrotic effects	Original article
4	Vitiligo	Pathogenesis of vitiligo and targeted treatments	Review
5	Psoriasis	Predictable blood biomarkers associated with liver fibrosis	Original article
6	Neutrophilic dermatosis	Treatment strategies and algorithms for management	Review
7	Non-melanoma skin cancer	In vitro study of a novel inhibitor with potential clinical applications	Original article
8	Skin drug reactions	Validation of the lymphocyte transformation test for the diagnosis of drug reactions	Original article
9	Non-melanoma skin cancer	Therapeutic local immunotherapy in premalignant conditions with imiquimod	Review
10	Psoriasis	Genetic influence on treatment response	Review
11	Non-melanoma skin cancer	In vitro study of a novel drug targeting p-53	Original research
